# Differential Effects of Turmeric Bioactive Compounds on Neuroinflammation and Mitochondrial Homeostasis in Brain Regions in a Rodent Model of Neuropathic Pain

**DOI:** 10.3390/metabo16070442

**Published:** 2026-06-25

**Authors:** Xiaobo Liu, Julianna M. Santos, Takaki Kiritoshi, Guangchen Ji, Volker Neugebauer, Chwan-Li Shen

**Affiliations:** 1 Department of Pathology, Texas Tech University Health Sciences Center, Lubbock, TX 79430, USA; 2 Department of Microanatomy and Cellular Biology, Woody L. Hunt School of Dental Medicine, Texas Tech University Health Sciences Center, El Paso, TX 79905, USA; 3 Department of Pharmacology and Neurosciences, Texas Tech University Health Science Center, Lubbock, TX 79430, USA; takaki.kiritoshi@ttuhsc.edu (T.K.);; 4 Garrison Institute on Aging, Texas Tech University Health Science Center, Lubbock, TX 79430, USA; 5 Center of Excellence for Translational Neuroscience and Therapeutics, Texas Tech University Health Science Center, Lubbock, TX 79430, USA; 6 Center of Excellence for Integrative Health, Texas Tech University Health Science Center, Lubbock, TX 79430, USA

**Keywords:** turmeric, neuropathy, neuroinflammation, mitochondria homeostasis, animals, brain

## Abstract

**Background:** Managing neuropathic pain (NP) is particularly challenging in the context of opioid use, and the mechanisms behind chronic pain remain unclear. **Objective:** This study evaluated the impact of turmeric bioactive compounds on brain regions including frontal cortex (FC), hippocampus (HPC), and hypothalamus (HPT) in the spinal nerve ligation (SNL) in a rat model of NP. **Methods:** Twenty-four SD rats were assigned to four groups (N = 6 per group), namely sham+vehicle (Sham-V), SNL+vehicle (SNL-V), SNL + 100 mg/kg curcumin (SNL+100CUR), and SNL + 50 mg/kg bisdemethoxycurcumin (SNL+50BDMC), treated daily for four weeks via oral gavage. Gene expression levels related to neuroinflammation, oxidative stress, and mitochondrial homeostasis were measured using qRT-PCR. Protein-level or functional mitochondrial assays were not performed due to limited sample availability. **Results:** In the FC, SNL decreased the expression level of NRF1 and OPA1, but only OPA1 was increased by BDMC. In the HPC, SNL increased CD11b, NRF2, and MFN1; BDMC decreased CD11b and increased IBA1, NRF1, TFAM, PGC1α and Complex I; and CUR increased NRF1, TFAM, DRP1 and Complex I levels. In the HPT, SNL decreased GFAP and MFN1, with CUR and BDMC further decreasing GFAP but not affecting MFN1. Additionally, CUR and BDMC decreased the expression of several key markers of neuroimmune signaling and mitochondrial homeostasis, including IBA1, CD11b, NFkB, NRF1/2, DRP1, OPA1, PGC1α, TFAM, and PINK1. **Conclusions:** CUR and BDMC induced region-specific transcriptional remodeling of mitochondrial homeostasis across FC, HPC, and HPT in SNL rats, with somewhat limited effects in the FC, mixed effects in the HPC, and broader downregulation in the HPT.

## 1. Introduction

Chronic pain poses a significant challenge to public health, affecting approximately one-fifth of the U.S. population [1]. Existing treatments, including opioids and non-steroidal anti-inflammatory drugs (NSAIDs), are often insufficient or only partially effective, and their use is linked to substantial adverse effects [2,3,4]. The development of more effective and safer therapeutic strategies requires a deeper understanding of the mechanisms underlying chronic pain.

Neuropathic pain (NP), caused by nerve injury, is a chronic condition shaped by persistent neuroinflammation, oxidative stress, and maladaptive neuroplasticity within the central nervous system (CNS) which promote neuronal sensitization and hyperexcitability [5,6,7,8]. Elevated reactive oxygen species (ROS) contributes to mitochondrial dysfunction during disease progression. Low-level central nervous system inflammation, glial dysregulation, and disrupted neuron–glia communication further exacerbate chronic NP by promoting central sensitization and heightened responsiveness to painful stimuli [5,7,8]. In light of this, strategies for reducing oxidative stress, proinflammatory cytokine release, and microglial activation are critical for mitigating CNS neuroinflammation and improving NP management [8,9].

Mitochondria-derived damage-associated molecular patterns (mtDAMPs) aggravate neuroinflammation and contribute to NP development [5,8,10]. Increasing evidence identifies mitochondrial dysfunction as a key regulator of sensory processing and pain pathophysiology [8]. Elevated levels of mitochondrial ROS production or mitochondrial Ca^2+^ can activate the NLR family pyrin domain containing 3 (NLRP3) inflammasome and/or recruit Parkin and PTEN-induced putative kinase 1 (PINK1) to damaged mitochondria, initiating mitophagy to remove dysfunctional organelles [8,11]. ROS-modified mitochondrial DNA further promotes pro-inflammatory IL-1β release via activation of the NLRP3 inflammasome during NP [8,12]. Persistent inflammation and oxidative stress also impair mitochondrial transport and disrupt quality control processes, including mitophagy, biogenesis, fusion, and fission, thereby exacerbating neuronal vulnerability [8]. Restoring mitochondrial function in sensory neurons has been shown to mitigate hyperalgesia in preclinical NP models [8,11].

Given NP’s role in the prevalence of chronic pain, there is a clear need for safe and effective tools to mitigate NP. Antioxidants targeting mitochondria exhibit therapeutic potential for numerous pathological conditions, including NP [13,14,15]. Over the past decade, bioactive compounds have gained increasing attention for NP management due to their ability to modulate peripheral and CNS signaling through anti-inflammatory and antioxidant mechanisms [16]. Among different bioactive compounds, the rhizome *Curcuma longa* (turmeric) and its principal component, curcumin, have a long history of anti-inflammatory, antioxidant, and anti-nociceptive use [8,17]. Recent reviews highlight robust anti-nociceptive effects of compounds derived from turmeric, particularly curcumin, in multiple NP animal models [16], namely modulating opioid receptor systems, suppressing glial activation, regulating synaptic transmission, and anti-inflammatory actions.

In this study, we did not conduct a de novo experimental screening of plant-derived turmeric bioactive compounds. Instead, our selection of Curcumin C3 Complex^®^ (CUR) and bisdemethoxycurcumin (BDMC) was based on extensive published evidence demonstrating their analgesic, anti-inflammatory, antioxidant, and neuroprotective activities relevant to neuropathic pain [18]. Curcuminoids have been shown to attenuate neuroinflammation, modulate oxidative stress pathways, and reduce neuropathic pain behaviors in multiple preclinical models [19,20,21,22,23]. CUR is a standardized preparation with well-characterized pharmacokinetics and safety characteristics [24], while BDMC exhibits superior chemical stability and potent anti-inflammatory and neuroprotective effects [25,26]. These literature-supported properties provided a strong mechanistic rationale for prioritizing CUR and BDMC as candidate agents for neuropathic pain.

We previously reported that turmeric extract and its bioactive constituents modulate glial activation, mitochondrial homeostasis (including mitophagy, biogenesis, fission, and fusion), and oxidative stress within the spinal cord and amygdala in a spinal nerve ligation (SNL) model of NP [8,27]. The spinal cord is part of the interface of peripheral central nociceptive processing while the amygdala plays an important role in emotional-affective pain and pain modulation [28,29]. However, it remains unknown whether turmeric’s modulatory effects on mitochondrial homeostasis affect other parts of the brain in animals with NP, such as the frontal cortex (FC) and hippocampus (HPC), which are elements of the corticolimbic system linked to chronic pain vulnerability [30,31], and the hypothalamus (HPT), a hub for autonomic and endocrine processes. The FC contributes to top-down modulation of pain and affective regulation, both of which are disrupted in NP [32,33,34]. The HPC integrates contextual memory and stress responses, and chronic NP is known to impair hippocampal plasticity and metabolic function [35,36]. NP is also associated with heightened HPA axis activation and inflammatory signaling [37,38], indicating that the HPT may be understudied in the context of pain mechanisms. Given their functions, these regions are important targets for evaluating mitochondrial homeostasis.

Assessing mitochondrial function in the FC, HPC, and HPT when treated with turmeric bioactive compounds provides critical insight into how these bioactive compounds alter corticolimbic and neuroendocrine networks of NP. To this end, our study evaluates the effects of turmeric extract, curcumin C3 Complex^®^ (CUR; 97% curcuminoids), and bisdemethoxycurcumin (BDMC; a bioactive curcuminoid) on glial activation, mitochondrial homeostasis, and oxidative stress in male rats across three brain regions, namely FC, HPC, and HPT, in an SNL-induced NP model [8]. We hypothesized that CUR and BDMC administration would attenuate glial activation and enhance mitochondrial function in the FC, HPC, and HPT of SNL animals. We expected that suppressed neuroinflammation and oxidative stress, along with improvements in antioxidant capacity and metabolic function, would lead to these outcomes.

Despite growing evidence that turmeric-derived bioactive compounds modulate inflammatory, oxidative, and mitochondrial pathways relevant to neuropathic pain, their region-specific effects at supraspinal levels remain poorly defined. Our previous work primarily characterized mechanisms of the spinal cord and amygdala in an SNL model [8], but other brain regions potentially involved in pain modulation, affective control, and neuroendocrine regulation remain to be explored. These include the frontal cortex (FC), hippocampus (HPC), and hypothalamus (HPT). To address this knowledge gap, the present study investigated whether CUR and BDMC differentially regulate neuroinflammatory, oxidative stress, and mitochondrial homeostasis-related transcriptional profiles across these supraspinal regions in the SNL model of NP. The novelty of this work lies in the direct comparison of CUR and BDMC and its brain region-specific molecular analysis, providing new insight into the supraspinal actions of turmeric-derived bioactive compounds.

## 2. Materials and Methods

### 2.1. Animals

We purchased twenty-four male Sprague Dawley rats (150–180 g) from Envigo (Cumberland, VA, USA) and housed them individually in a 12-h light–dark cycle. Water and food were administered ad libitum. The Institutional Animal Care and Use Committee at Texas Tech University Health Sciences Center (IACUC #21007) approved all procedures which we conducted according to all relevant guidelines and regulations.

This study utilized a subset of brain tissues collected from the same cohort of male rats used in our previously published SNL-induced NP study, and NP-influenced behavior was established previously in the animals from which tissue for the present study was obtained [8]. Male rats were used to maintain methodological consistency with the original experimental design and to minimize biological variability associated with the estrous cycle in female rats, which can influence pain sensitivity and neuroimmune responses [39,40,41]. All animals, surgical procedures, and tissue collection protocols were identical to those described in Santos, 2023 [8].

### 2.2. Induction of Neuropathic Pain

After a 5-day acclimatization period, animals were assigned to experimental groups based on body weight to balance the mean body weight across groups. We randomly assigned six animals to the sham-control group and performed sham surgery, while the other eighteen animals underwent SNL procedures. The SNL model induces NP in the left hind paw, as we have previously outlined [8,42,43]. We induced anesthesia briefly with 3% isoflurane and maintained 2% throughout the procedure. Following removal of the L5/L6 paraspinal muscles and the underlying L6 transverse process, we isolated the L5 spinal nerve from neighboring tissues and tightly ligated it with 6-0 silk suture. The paraspinal muscles were then sutured and the skin was secured with wound clips. Sham-operated animals underwent identical surgical exposure without spinal nerve ligation. Postoperatively, we administered a single dose of gentamicin (8 mg/kg, subcutaneous; VetOne, Boise, ID, USA) to all animals and monitored them for signs of infection or distress [8]. Animals were observed longitudinally to minimize unnecessary pain or stress, in accordance with ethical guidelines from the International Association for the Study of Pain [44]. We assessed mechanical pain via the von Frey test (Electronic von Frey Aesthesiometer, IITC Life Science, Woodland Hills, CA, USA) as described in the previous study [8].

### 2.3. Dietary Treatments

Turmeric extract contains three principal curcuminoids, curcumin (≈80% relative abundance), demethoxycurcumin (≈15%), and bisdemethoxycurcumin (BDMC; ≈5%), which differ in their methoxy substitutions on the aromatic ring [45]. Curcumin C3 Complex^®^ (CUR) is a standardized preparation containing a defined ratio of these three curcuminoids and has Generally Recognized as Safe (GRAS) status. Both BDMC and demethoxycurcumin help keep curcumin stable and enhance its gastrointestinal absorption [46]. We selected purified BDMC for comparison with CUR in this NP model for two reasons: (i) BDMC is more chemically stable than curcumin and demethoxycurcumin and (ii) BDMC exhibits multiple pharmacological activities, including antioxidant [47], anti-inflammatory [48], pro-apoptotic [49], analgesic [50], and neuroprotective effects [8,51]. BDMC’s multi-mechanistic profile supports its potential therapeutic efficacy across several pathophysiological processes relevant to NP.

We randomly assigned our 24 rats to four treatment groups: Sham-V, SNL-V, SNL+CUR at 100 mg/kg BW (SNL+100CUR), and SNL+BDMC at 50 mg/kg BW (SNL+50BDMC). We fed all animals the AIN-93G diet (catalog #D10012G, Research Diets, Inc., New Brunswick, NJ, USA). We evaluated CUR at 25, 50, and 100 mg/kg BW dosages, p.o. and BDMC at 50 mg/kg BW, and observed no significant difference in mechanical hypersensitivity between SNL+100CUR and SNL+50BDMC rats [8]. Therefore, we selected the latter two doses for this study. Using the standard body-surface-area method for interspecies dose translation, our doses of 100 mg/kg CUR and 50 mg/kg BDMC in rats correspond to human equivalent doses of approximately 16.2 and 8.1 mg/kg, respectively, which translate to about 1 g/day CUR and 0.5 g/day BDMC for a 60–70 kg adult [52]. These values fall within the range of curcumin doses (~500–2000 mg/day) that have been widely used and shown to be safe and pharmacologically active in clinical studies of pain and peripheral neuropathies [24,53,54], thereby supporting the translational relevance of our chosen preclinical doses.

Animals in the SNL+100CUR and SNL+50BDMC groups received their dosages over the course of four weeks starting when we performed SNL surgery. Both CUR and BDMC (>99% purity, derived from turmeric extract) were provided by Sabinsa Corporation (East Windsor, NJ, USA). The CUR preparation contained 97.34% total curcuminoids, including 77.3% curcumin, 19.0% demethoxycurcumin, and 3.7% BDMC. We monitored body weight, food intake, and water consumption weekly [8].

### 2.4. Sample Collection

At the end of the experiment, animals were anesthetized with isoflurane prior to euthanasia, and the designated brain regions were rapidly dissected, flash-frozen in liquid nitrogen, and stored at −80 °C for subsequent mRNA expression analysis.

### 2.5. RNA Isolation and qRT-PCR

We isolated total RNA from the FC, HPC, and HPT using reagents RNAzol RT (RN190) and BAN (BN191) (Molecular Research Center Inc., Cincinnati, OH, USA) at a 1:200 ratio. We measured RNA concentration and purity using a NanoDrop spectrophotometer at 260 nm (NanoDrop One, Thermo Scientific, Dallas, TX, USA) and synthesized cDNA from total RNA using a thermal cycler (Bio-Rad S1000, Bio-Rad Laboratories, Hercules, CA, USA). We performed quantitative real-time PCR (qRT-PCR) on a QuantStudio 12K Flex system (Life Technologies, Carlsbad, CA, USA) using Universal SYBR Green Supermix (Bio-Rad Laboratories, 17251-24) to amplify target genes (Table 1). Relative gene expression was calculated using 2^−(ΔCT × 1000)^ values normalized to β-actin, as described previously [55]. Supplementary File S1 provides additional methodological details, including amplification data, raw result data, and melt-curve raw data of each brain region.

### 2.6. Statistical Analysis

Blinding was not possible due to the apparent phenotypic differences between SNL and sham animals. Data are given as the mean ± standard error of the mean (SEM). The mRNA expression data were analyzed using one-way ANOVA followed by Tukey’s post hoc tests. We conducted all analyses with GraphPad Prism 10 (GraphPad Software, San Diego, CA, USA). Statistical significance was set at *p* < 0.05, and results with 0.05 < *p* < 0.1 were reported as trend #.

## 3. Results

### 3.1. Turmeric Supplementation Changed mRNA Expression Level of Microglial and Astrocyte Activation, and Inflammation

**Figure 1:**
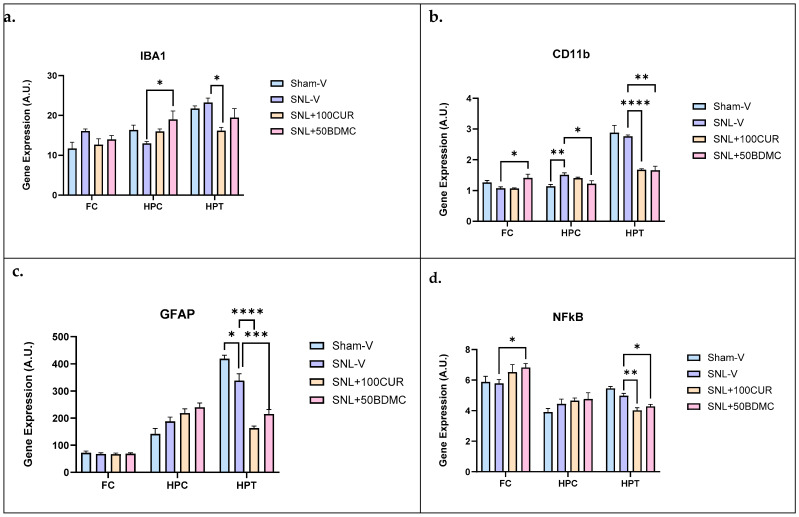
Effect of turmeric bioactive compound supplementation on IBA-1 (**a**), CD11b (**b**), GFAP (**c**), and NFκB (**d**) mRNA expression in the FC, HPC, and HPT of SNL rats. Data are presented as mean ± SEM. The four experimental groups included Sham-V, SNL-V, SNL+100CUR (100 mg curcumin C3 Complex^®^/kg BW daily), and SNL+50BDMC (50 mg bisdemethoxycurcumin/kg BW daily), with *n* = 5–6 animals per group. Within each brain region, data were analyzed by one-way ANOVA followed by Tukey’s post hoc test. * *p* < 0.05, ** *p* < 0.01, *** *p* < 0.001, **** *p* < 0.0001.

We measured CUR/BDMC’s effect on gene expression (mRNA levels) of microglial activation markers [IBA-1 (Figure 1a) and CD11b (Figure 1b)], the astrocyte activation marker [GFAP (Figure 1c)], and transcription factor NFκB, which is an immune and inflammation regulator (Figure 1d), in FC, HPC, and HPT. Neither CUR nor BDMC altered IBA1 gene expression in the FC of SNL-V verses Sham-V group. In the HPC, no differences were found between Sham-V and SNL-V but the SNL+50BDMC group showed markedly higher IBA1 gene expression compared with the SNL-V group. In the HPT, no difference was found between Sham-V and SNL-V, but the SNL+100CUR group exhibited lower IBA1 gene expression than the SNL-V group (Figure 1a).

Compared to the Sham-V group, the SNL-V group showed increased CD11b mRNA expression levels in the HPC, but not in the FC or HPT. Relative to the SNL group, the SNL+50BDMC group showed increased CD11b mRNA levels in the FC, but decreased CD11b mRNA expression in both HPC and HPT. In the HPT, CUR (100 mg/kg) also decreased CD11b expression compared with the SNL-V group (Figure 1b).

Compared to the Sham-V group, neither SNL+100CUR nor SNL+50BDMC showed altered GFAP gene expression levels in the FC or HPC. In the HPC, we observed that (i) the SNL-V group showed lower GFAP gene expression than the Sham-V group, and (ii) both SNL+100CUR and SNL+50BDMC groups showed further reductions in GFAP gene expression (Figure 1c). The SNL procedure did not alter NFκB expression in any of the examined brain regions. NFκB gene expression levels were elevated in the SNL+50BDMC group in the FC compared to the SNL group. Among the three regions studied, CUR and BDMC administration only significantly suppressed NFκB expression in the HPT of the SNL-V group.

### 3.2. Turmeric Bioactive Compound Supplementation Altered mRNA Expression of Mitochondrial Fusion, Fission, and Biogenesis Markers

**Figure 2:**
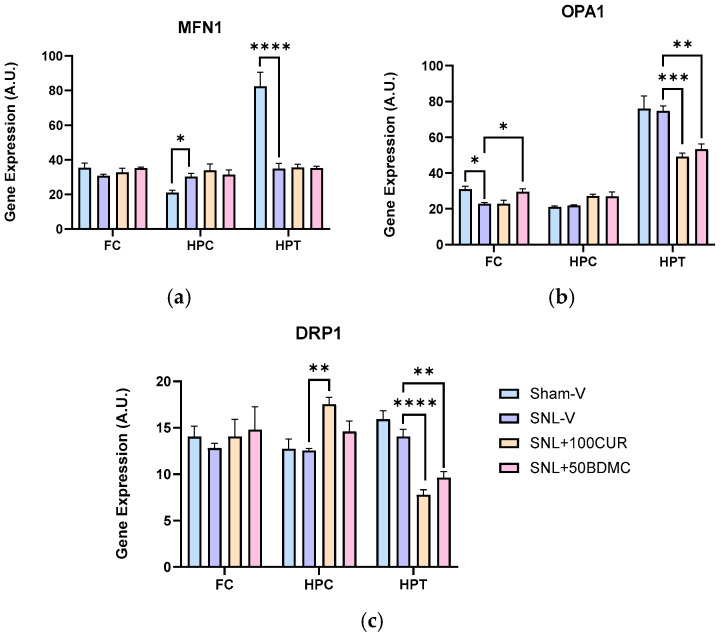
Effect of turmeric bioactive compound supplementation on MFN1 (**a**), OPA1 (**b**), and DRP1 (**c**) mRNA expression in the FC, HPC, and HPT of SNL rats. Data are presented as mean ± SEM. The four experimental groups included Sham, SNL, SNL+100CUR (100 mg curcumin C3 Complex^®^/kg BW daily), and SNL+50BDMC (50 mg bisdemethoxycurcumin/kg BW daily), with *n* = 5–6 animals per group. Within each brain region, data were analyzed by one-way ANOVA followed by Tukey’s post hoc test. * *p* < 0.05, ** *p* < 0.01, *** *p* < 0.001, **** *p* < 0.0001.

We examined the effects of turmeric bioactive compound supplementation on MFN1 (Figure 2a) and OPA1 (Figure 2b), markers of mitochondrial fusion, and DRP1 (Figure 2c), a mitochondrial fission marker, in the FC, HPC, and HPT of SNL rats. Relative to the Sham-V group, rats in the SNL-V group showed increased MFN1 expression in the HPC but decreased MFN1 expression in the HPT. Neither SNL+100CUR nor SNL+50BDMC group showed altered MFN1 expression in the FC, HPC, or HPT (Figure 2a). The SNL+50BDMC group showed increased OPA1 in FC that was decreased in the SNL-V group and both SNL+100CUR and SNL+50BDMC groups showed significantly suppressed OPA1 expression in the HPT, but not in the HPC, of SNL-V group (Figure 2b). The SNL-V group had reduced OPA 1 expression level in FC, which was reversed in the SNL+50BDMC group. The SNL+100CUR and SNL+50BDMC groups also showed reduced DRP1 expression in the HPT. Additionally, the SNL+100CUR group exhibited significantly elevated DRP1 expression in the HPC compared with the SNL-V group (Figure 2c).

**Figure 3:**
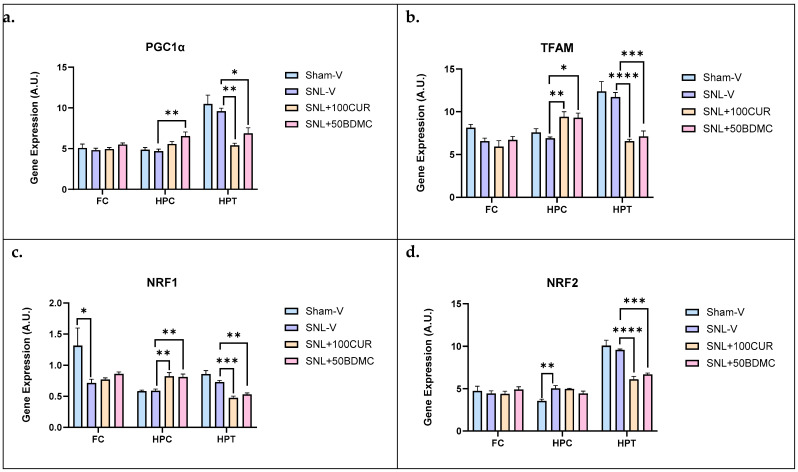
Effects of turmeric bioactive compound supplementation on gene expression of PCG1α (**a**), TFAM (**b**), NRF1 (**c**) and NRF2 (**d**) in the FC, HPC, and HPT of SNL rats. Data is expressed as mean ± SEM. Data are presented as mean ± SEM. The four experimental groups included Sham, SNL, SNL+100CUR (100 mg curcumin C3 Complex^®^/kg BW daily), and SNL+50BDMC (50 mg bisdemethoxycurcumin/kg BW daily), with *n* = 5–6 animals per group. Within each brain region, data were analyzed by one-way ANOVA followed by Tukey’s post hoc test. * *p* < 0.05, ** *p* < 0.01, *** *p* < 0.001, **** *p* < 0.0001.

We measured the effects of turmeric bioactive compound supplementation on PGC1α (Figure 3a), a key regulator of mitochondrial biogenesis, and TFAM (Figure 3b), a marker of mitochondrial function, in the FC, HPC, and HPT of rats with SNL. We observed no significant differences in PGC1α or TFAM expression between the Sham-V and SNL-V groups across the three brain regions. BDMC supplementation increased PGC1α expression in the HPC but decreased its expression in the HPT relative to the SNL-V group (Figure 3a). CUR supplementation decreased PGC1α expression level in the HPT relative to the SNL-V group. Both SNL+100CUR and SNL+50BDMC groups showed elevated expression of TFAM in the HPC while suppressing TFAM in the HPT of SNL-V group (Figure 3b).

We measured the effects of turmeric bioactive compound supplementation on gene expression of NRF1 (Figure 3c) and NRF2 (Figure 3d), which are regulators of antioxidant genes supporting mitochondrial biogenesis. In contrast to the Sham-V group, the SNL-V group suppressed NRF1 gene expression in the FC. Both SNL+100CUR and SNL+50BDMC group increased NRF1 gene expression in the HPC, while they decreased NRF1 gene expression in the HPT (Figure 3c). In contrast, the SNL-V group showed significantly elevated NRF2 expression in the HPC. In the HPT, both SNL+100CUR and SNL+50BDMC groups showed suppressed NRF1 and NRF2 expression (Figure 3d).

### 3.3. Turmeric Bioactive Compounds Supplementation Altered mRNA Expression of Mitochondrial Electron Transport Chain, Oxidative Stress, and Autophagy

**Figure 4:**
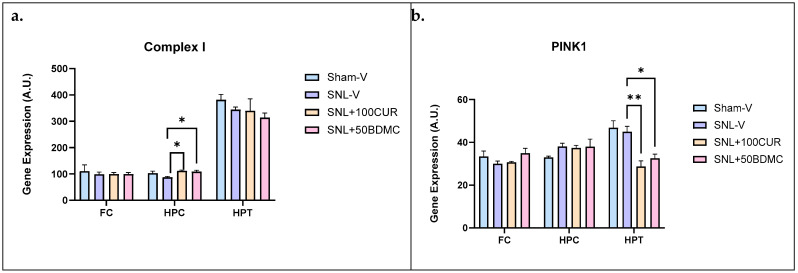
Effects of turmeric bioactive compound supplementation on gene expression of Complex I (**a**) and PINK1, a mitochondrial kinase (**b**), in the FC, HPC, and HPT of SNL rats. Data are presented as mean ± SEM. The four experimental groups included Sham, SNL, SNL+100CUR (100 mg curcumin C3 Complex^®^/kg BW daily), and SNL+50BDMC (50 mg bisdemethoxycurcumin/kg BW daily), with *n* = 5–6 animals per group. Within each brain region, data were analyzed by one-way ANOVA followed by Tukey’s post hoc test. * *p* < 0.05, ** *p* < 0.01.

We evaluated the effects of turmeric bioactive compounds on expression of complex I gene, a component of the mitochondrial respiratory chain (Figure 4a), and expression of PINK1 gene (Figure 4b), a marker of mitochondrial autophagy, in rats with SNL. Both SNL+100CUR and SNL+50BDMC groups showed markedly increased complex I expression in the HPC, with no changes observed in the FC or HPT (Figure 4a). PINK1 expression in the FC and HPC was not altered either in SNL-V or SNL+100CUR and SNL+50BDMC groups. In contrast, both SNL+100CUR and SNL+50BDMC groups showed significantly suppressed expression of PINK1 in the HPT of the rats (Figure 4b).

## 4. Discussion

Our study is the first to characterize the region-specific transcriptional effects of Curcumin C3 Complex^®^ (CUR) and BDMC on markers of glial activation, inflammation, and mitochondrial homeostasis within the FC, HPC, and HPT of SNL rats. Our principal finding reveals a non-uniform supraspinal response to turmeric bioactives marked by significant regional divergence. While the FC remained largely unresponsive at the four-week time point, the HPC and HPT exhibited robust but often opposing transcriptional shifts. Such regional selectivity supports the broader conclusion that supraspinal responses to SNL and nutritional interventions are governed by distinct tissue-specific regulatory programs, potentially dictated by the unique metabolic and physiological demands of each region: the HPC in cognitive-affective processing and the HPT in neuroendocrine and autonomic integration [6,37].

In the FC, we observed a relatively muted response except for isolated SNL-associated reductions in NRF1 and OPA1 (Figure 2b and Figure 3c), suggesting disruption of mitochondrial maintenance and fusion-related signaling under neuropathic stress. NRF1 is an important regulator of mitochondrial biogenesis and respiratory gene expression [56,57], while OPA1 is essential for mitochondrial fusion, cristae organization, and maintenance of bioenergetic integrity [58,59]. Mitochondrial dysfunction is key in the development of neuropathic pain, as the imbalanced energy production can trigger apoptosis and activate inflammatory pathways, contributing to hyperalgesia [60]. BDMC reversed the decrease in OPA1, which may indicate partial restoration of mitochondrial fusion-related homeostasis. This result corroborates our previous study that BDMC mitigates SNL-induced OPA1 decrease in the amygdala [27]. However, neither nerve injury nor turmeric supplementation markedly altered most transcripts. This suggests the FC may be less transcriptionally responsive than the HPC or HPT at this specific time point, perhaps due to a more resilient blood–brain barrier (BBB) or delayed onset of neuroinflammatory remodeling compared to limbic and homeostatic centers. However, these were not directly evaluated in the present study, so a future validation study is needed for conclusive determinations.

By contrast; the HPC and HPT showed pronounced but distinct neuroinflammatory profiles at the transcriptional level. Microglial and astrocytic activation are central to the pathogenesis of neuropathic pain (NP), contributing to a state of chronic central sensitization [61]. In this context, evaluating IBA 1, CD11b, GFAP, and NFκB expression in the HPC and HPT is essential for understanding how turmeric bio-actives mitigate NP induced central neuroinflammation. SNL increased the microglial marker CD11b in the HPC (Figure 1b), a shift associated with early neuroimmune responses [62,63], which only BDMC effectively attenuated. This enhanced efficacy is consistent with reports of BDMC’s greater metabolic stability and superior bioavailability relative to curcumin [64]. Specifically, the absence of methoxy groups in BDMC may reduce its rate of glucuronidation, allowing higher active concentrations to reach the hippocampal formation. Moreover, BDMC’s suppression of CD11b aligns with prior reports showing that curcumin reduces hippocampal IL 1β and TNFα levels in the CCI model of NP [65,66], reinforcing the beneficial role of turmeric bioactives in inhibiting hippocampal neuroinflammation. However, the finding that BDMC increased IBA1 while both compounds further reduced GFAP highlights the complexity of glial remodeling; since we did not directly assess microglial morphology, cell-specific marker expression, or functional phenotype, we hypothesized that turmeric bioactives appear to induce a differential shift in activation states, potentially moving microglia toward a more surveillance-oriented phenotype rather than a simple “normalization” of the pathologic milieu [67]. NP is known to impair hippocampal-dependent cognitive functions and exacerbate emotional disturbances [65,66]. The SNL procedure used in this study reduces neurogenesis and synaptic plasticity in the HPC, creating a milieu in which microglial activation is particularly consequential [68]. Interestingly, BDMC decreased CD11b while increasing IBA1 in the same region, suggesting that these microglia-associated markers may reflect distinct aspects of the neuroimmune response. CD11b is associated with integrin-mediated adhesion, migration, and inflammatory immune activation, whereas IBA1 is involved in actin remodeling and microglial morphological dynamics. Thus, BDMC may not simply suppress microglial activation globally but may instead alter the balance of microglia-related transcriptional programs in the hippocampus. However, because the present study did not assess microglial morphology, cell-specific expression, or protein-level activation markers, this interpretation remains a knowledge gap that calls for validation by immunohistochemistry or cell-specific analyses.

Conversely, the HPT demonstrated a robust and uniform suppression of all measured neuroinflammatory markers, including IBA1, CD11b, GFAP, and NFκB (Figure 1a–d). The HPT integrates systemic stress signals and possesses specialized vascular regions with higher permeability; this may facilitate greater penetration of curcuminoids, allowing for effective suppression of NFκB-mediated cytokine production [8,69]. Because HPT neuroinflammation disrupts neuroendocrine and autonomic integration, this suppression suggests a significant dampening of stress-related signaling in this homeostatic center [6,37]. The broad downregulation observed in the hypothalamus may reflect a region-specific transcriptional response to CUR or BDMC. Whether this represents adaptive dampening, reduced inflammatory tone, or another compensatory mechanism remains to be determined.

Mitochondrial dysfunction and oxidative stress reduce spinal inhibitory transmission and drive maladaptive synaptic plasticity [70,71,72]. CUR and BDMC exerted divergent effects on the PGC1α/TFAM axis, the master regulatory pathway of mitochondrial biogenesis [73]. PGC1α is a central transcriptional coactivator that regulates cellular energy metabolism and serves as the master regulator of mitochondrial biogenesis. It helps maintain redox balance during neuroinflammation by promoting antioxidant gene expression [73]. PGC1α expression and activity are tightly regulated by cytokines, transcription factors such as TFAM, and diverse external stimuli through several intracellular signaling pathways [74]. As a master regulator of mitochondrial biogenesis and cellular energy metabolism, PGC1α responds dynamically to inflammatory and metabolic cues. Our study is the first to show how CUR and BDMC exert region-specific effects on mitochondrial biogenesis markers in the CNS. The reduction in Complex I gene expression in the HPC following SNL conforms to past findings showing that NP impairs mitochondrial respiration, including deficits in Complex I- and Complex III-mediated oxidative phosphorylation in sciatic nerve isolated mitochondria [8,75]. Mitochondria generate ATP through oxidative phosphorylation, a process driven by the coordinated activity of the mitochondrial electron transport chain (mt ETC) complexes I–IV. While these redox reactions are essential for ATP production, they also generate ROS as by products. Thus, decreased Complex I expression in the HPC of SNL rats suggests compromised mt ETC function and a potential shift toward oxidative stress. In the HPC, both compounds increased the expression of PGC1α, TFAM, and Complex I (Figure 3a,b and Figure 4a). Given that NP impairs hippocampal neurogenesis and plasticity, these increases may reflect “bioenergetic priming”. By upregulating biogenic markers, turmeric bioactive may provide the metabolic support necessary to preserve energy metabolism and support synaptic remodeling in a region highly vulnerable to chronic pain-induced atrophy [66,68,76]. Future studies are required for conclusive determinations.

In the HPT, however, CUR and BDMC broadly reduced transcripts related to biogenesis (PGC1α, TFAM, NRF1/2), dynamics (DRP1, OPA1), and mitophagy (PINK1). This coordinated downregulation of mitochondrial turnover, specifically the reduction in the fission marker DRP1, suggests that turmeric bioactives may stabilize mitochondrial structure and reduce the cellular requirement for compensatory cycling [8,13]. The suppression of PINK1, a mitochondrial stress sensor [77], further supports the interpretation that curcuminoids alleviate upstream oxidative burden, thereby reducing the need for mitophagic clearance [78]. These mitochondrial-protective actions are likely facilitated by the lipophilic nature of these compounds, allowing them to interact directly with high-metabolic demand tissues [79]. PINK1 (PTEN induced putative kinase 1) is highly expressed in neurons and serves as a key mitochondrial stress sensor kinase, particularly in brain regions with high metabolic demand such as the hippocampus, cerebellum, and dopaminergic systems [77]. Under conditions of mitochondrial depolarization or oxidative injury, PINK1 accumulates on the outer mitochondrial membrane, where it initiates mitophagy. Notably, PINK1 expression is enriched in GABAergic interneurons, and co-localization studies have shown that PINK1 is selectively upregulated within autophagic mitochondria, as evidenced by its overlap with BECN1, LC3 II, and COX IV [78]. These findings underscore PINK1’s potential role in identifying damaged mitochondria and triggering their removal through the mitophagy pathway. A major limitation is that the molecular analyses were performed at the mRNA level only. The abundance and activity of mitochondrial regulatory proteins, including OPA1, DRP1, and PINK1, are strongly influenced by post-translational modifications, proteolytic processing, and subcellular localization. Therefore, the observed transcriptional changes cannot be assumed to directly reflect protein abundance or mitochondrial function. Future studies should validate key findings at the protein level using Western blotting or immunohistochemistry and should include functional mitochondrial assays, particularly in the hypothalamus, where the most pronounced transcriptional response was observed.

A notable feature of this dataset was the opposite directional response observed between the HPC and HPT. These opposing patterns likely reflect the distinct physiological roles of these regions: the HPC manages high-demand synaptic plasticity and cognitive processing, while the HPT serves to monitor homeostatic and stress responses [37,80]. In the HPC, elevated NRF1/NRF2 and PGC1α levels may represent a “pro-active” strategy to prioritize metabolic support [81]. In the HPT, the broad downregulation may reflect an “adaptive dampening” of pathological over-activation, effectively resetting the transcriptional tone of this homeostatic hub to prevent chronic sympathetic “noise” [82,83]. Support for such differential mitochondrial regulation of HPC and HPT comes from prior studies of curcumin derivatives. Vacek et al. (2018) [84] reported that tetrahydrocurcumin improved mitochondrial remodeling in brain endothelial cells by reducing DRP1, MFN2, and the autophagy marker LC3, indicating broad effects on mitochondrial quality control pathways [84]. Similarly, Zhai et al. showed that curcumin elevated PGC 1α expression in hepatic stellate cells by activating AMP-activated protein kinase, a key cellular energy sensor [8,85]. These findings highlight the capacity of curcumin-related compounds to modulate mitochondrial biogenesis and turnover across diverse tissues. Curcuminoids are lipophilic compounds capable of crossing the highly selective BBB, allowing them to directly interact with CNS tissues. Although nanoparticle formulations have been developed to further enhance their bioavailability [79], native curcuminoids already demonstrate favorable brain permeability. This property may help explain why both CUR and BDMC modulated mitochondria-associated gene expression in HPC and HPT of SNL-treated rats, which requires assessment by further studies.

NRF1 and NRF2 play distinct yet complementary roles in NP, with NRF1 regulating mitochondrial function and NRF2 coordinating antioxidant defenses [83]. Together, NRF1 and NRF2 help prevent the cycle of nerve injury, mitochondrial dysfunction, and neuroinflammation characteristic of NP. In this study, CUR and BDMC produced opposite effects on NRF1 and NRF2 expression in the HPC (increased) and HPT (decreased) of SNL-treated rats (Figure 3c,d), mirroring the region-specific patterns observed for PGC 1α and TFAM (Figure 3a,b). These opposing responses likely reflect the different physiological roles of the HPC and HPT in managing mitochondrial stress, oxidative load, and proteasome activity rather than simple compensatory regulation. Prior studies show that NRF1 and NRF2 can function on opposite sides of a cellular stress response balance: elevated NRF1 may coincide with reduced NRF2 activation when cells prioritize proteasome maintenance over antioxidant defense, and vice versa [82,86]. NRF1 can even repress NRF2-mediated transcription under certain conditions. Although both factors bind antioxidant response elements, their regulatory mechanisms diverge, leading to region-specific gene activation patterns in specialized tissues such as the HPC and HPT [82,83,86]. Although this study evaluated oxidative stress- and mitochondrial homeostasis-related markers at the transcriptional level, direct measurements of cytosolic ROS and mitochondrial ROS were not performed due to limited tissue availability. Functional ROS assays, including total cellular ROS and mitochondrial superoxide measurements (e.g., DCFDA-based cytosolic ROS detection) and mitochondrial superoxide assessment (e.g., MitoSOX-based mitochondrial ROS measurement), are needed to determine whether CUR and BDMC directly attenuate oxidative stress in NP-related tissues.

Importantly, many treatment effects in the HPT represented shifts to levels below the physiological baseline rather than a simple reversal of SNL-induced increases. These findings must be interpreted with caution, as measurements were performed in bulk tissue at the mRNA level, which may not fully reflect protein activity or mitochondrial flux. Therefore, these results should be interpreted as evidence of region-specific transcriptional remodeling rather than definitive functional recovery. This heterogeneity may explain why nutritional interventions in chronic pain often yield complex molecular outcomes even when behavioral benefits are observed [8]. Collectively, our results demonstrate that CUR and BDMC mitigate central neuroinflammation and stabilize mitochondrial dynamics through multiple, tissue-specific mechanisms, reinforcing their potential as multi-target therapeutic strategies for neuropathic pain, with BDMC showing particular stability and efficacy in the HPC.

## 5. Study Limitations

Our findings are subject to five notable limitations. First, due to the limited tissue volume available from the FC, HPC, and HPT, all outcomes were assessed exclusively at the transcriptional level. The lack of protein-level validation (e.g., Western blotting or immunohistochemistry) or functional assays limits our ability to draw definitive conclusions regarding post-transcriptional regulation, protein activity, or overall pathway flux. This is particularly relevant for interpreting the broad downregulation observed in the HPT, as its functional significance cannot be determined from the present transcriptomic dataset alone. It remains to be determined if and how the observed mRNA-level changes translate into the corresponding protein expression of key markers. Second, because analyses were performed on bulk tissue samples, we were unable to identify cell-type-specific contributions to the observed neuroinflammatory and mitochondrial responses. In addition, this study did not directly assess inflammasome activation. Given the established roles of NLRP3 and NLRP6 inflammasomes in neuroinflammation and immune regulation [87,88,89], this knowledge gap can be addressed by evaluating inflammasome-related markers, including NLRP3, NLRP6, ASC, cleaved caspase-1, IL-1β, and IL-18, to determine whether inflammasome modulation contributes to the effects of turmeric-derived bioactive compounds in NP. Third, this study examined only a single post-injury time point; therefore, it remains unclear whether the regional responses reflect transient adaptations or sustained remodeling during the progression of NP. Fourth, although neuronal activation markers were not assessed in this study due to limited tissue availability, prior research has demonstrated that curcumin and BDMC modulate neuronal activity and neuroplasticity. Curcuminoids have been shown to reduce c-Fos expression in nociceptive pathways [22,90], enhance neuronal survival as indicated by increased NeuN-positive neurons [14,91,92], and upregulate neuroplasticity-related markers such as BDNF, GAP-43, and synaptic proteins including synapsin-1 and PSD-95 [14,93,94]. These published findings support the potential of turmeric bioactive compounds to influence neuronal function in NP-related brain regions, warranting future studies incorporating direct neuronal marker analyses. Finally, this study analyzed tissues derived exclusively from male rats in the previously published [8] cohort and was not designed to test for potential sex-specific differences. Given well-documented sex differences in pain processing, neuroinflammation, and mitochondrial function [39,40], future studies incorporating female cohorts will be needed to determine whether CUR and BDMC exert sex-dependent effects. Such studies, alongside larger-scale protein and single-cell transcriptomic analyses, will be necessary to determine if CUR and BDMC exhibit sex-specific effects and to facilitate the translation of turmeric-derived bioactives into broadly applicable therapeutic strategies.

## 6. Conclusions

In conclusion, CUR and BDMC elicited distinct, brain-region-specific transcriptional responses in the SNL-induced neuropathic pain model. Among the regions examined, the hypothalamus exhibited the most pronounced transcriptional changes following treatment with turmeric-derived bioactive compounds. These findings indicate that CUR and BDMC may differentially modulate neuroinflammatory, oxidative stress, and mitochondrial homeostasis-related pathways at the transcriptional level. Future studies incorporating protein-level validation, histological analyses, behavioral assessments, and mitochondrial functional assays are needed to determine the mechanism and significance of these region-specific molecular effects in neuropathic pain.

## Figures and Tables

**Table 1:** List of primers for mRNA [8].

Gene	Forward	Reverse
IBA1	5′-GAG CTA TGA GCC AGA GCA AGG ATT T-3′	5′-ACT CCA TGT ACT TCG TCT TGA AGG-3′
CD11b	5′-TCC AAC CTG CTG AGG AAG CC-3′	5′-TCG ATC GTG TTG ATG CTA CCG-3′
GFAP	5′-AAT CTC ACA CAG GAC CTC GGC-3′	5′-AGC CAA GGT GGC TTC ATC CG-3′
NF-kB	5′-CCT CCA CCC CGA CGT ATT GC-3′	5′-GCC AAG GCC TGG TTT GAG AT-3′
MFN1	5′-AGC TCG CTG TCA TTG GGG AG-3′	5′-TCC CTC CAC ACT CAG GAA GC-3′
OPA1	5′-CAG CTG GCA GAA GAT CTC AAG-3′	5′-CAT GAG CAG GAT TTT GAC ACC-3′
DRP1	5′-ACA ACA GGA GAA GAA AAT GGA GTT G-3′	5′-AGA TGG ATT GGC TCA GGG CT-3′
PGC1α	5′-CAG GAG CTG GAT GGC TTG GG-3′	5′-GGG CAA AGA GGC TGG TCC T-3′
TFAM	5′-GCT TCC AGG GGG CTA AGG ATG-3′	5′-TCG CCC AAC TTC AGC CAT TT-3′
NRF1	5′-AGC AGC CGT TGG AGC ACT TA-3′	5′-CGT CAC GGC TTT GCT GAT GG-3′
NRF2	5′-CTC TCT GGA GAC GGC CAT GAC-3′	5′-CTG GGC TGG GGA CAG TGG TAG T-3′
Complex I	5′-GGT TTG TCT ACA TCG GCT TCC-3′	5′-TAC AGA AGC TGG CGA TGC AAA-3′
PINK1	5′-TCG GCC TGT CAG GAG ATC CA-3′	5′-CAT TGC AGC CCT TGC CGA TG-3′
β- actin	5′-ACA ACC TTC TTG CAG CTC CTC C-3′	5′-TGA CCC ATA CCC ACC ATC ACA-3′

Abbreviations: IBA1, allograft inflammatory factor 1; CD11b, cluster of differentiation molecule 11B; GFAP, glial fibrillary acidic protein; NF-kB; MFN1, mitofusin 1; OPA1, optic Atrophy 1; DRP1, dynamin-related protein 1; PGC1α, also known as peroxisome proliferator-activated receptor gamma coactivator 1-alpha (Ppargc1α); TFAM, mitochondrial transcription factor A; NRF1, nuclear respiratory factor 1; NRF2, nuclear respiratory factor 2; PINK1, PTEN-induced kinase 1.

## Data Availability

The original contributions presented in this study are included in the article/Supplementary Material. Further inquiries can be directed to the corresponding author.

## Supplementary Materials

The following supporting information can be downloaded at: https://www.mdpi.com/article/10.3390/metabo16070442/s1, File S1: 31 October 2023 143718 HPT PGC1a, OPA1, TFAM, PINK1, ComplexI, NRF2_QuantStudio 12K Flex_export; File S2: 1 November 2023 105830 HPT NRF1, NFkB, GFAP, CD11b, IBA1, DRP1, MFN1_QuantStudio 12K Flex_export; File S3: 8 November 2023 102709 HPC NRF1, NFkB, GFAP, CD11b, IBA1, DRP1, MFN1_QuantStudio 12K Flex_export; File S4: 8 November 2023 141657 HPC PGC1a, OPA1, TFAM, PINK1, ComplexI, NRF2_QuantStudio 12K Flex_export; File S5: 20 November 2023 140255 FC Curcumin NRF1, NFkB, GFAP, CD11B, IBA1, DRP1, MFN1_QuantStudio 12K Flex_export; File S6: 21 November 2023 140139 FC PGC1a, OPA1, TFAM, PINK1, ComplexI, NRF2_QuantStudio 12K Flex_export.
